# Recent Advances in the Therapeutic Potential of Cannabinoids Against Gliomas: A Systematic Review (2022–2025)

**DOI:** 10.1002/prp2.70160

**Published:** 2025-08-08

**Authors:** Farideh A. Javid, Andrej Belancic, Man Ki Kwok, Yun Wah Lam

**Affiliations:** ^1^ Department of Pharmacy School of Applied Sciences, University of Huddersfield Huddersfield UK; ^2^ Department of Basic and Clinical Pharmacology With Toxicology, Faculty of Medicine University of Rijeka Rijeka Croatia; ^3^ Department of Health Sciences School of Nursing and Health Sciences, Hong Kong Metropolitan University Hong Kong SAR China

**Keywords:** cannabinoid therapeutics, endocannabinoid signaling, glioblastoma, glioma, systematic review

## Abstract

Glioma is the most common and lethal primary brain tumor in adults, with glioblastoma (GBM) representing the most aggressive subtype, characterized by diffuse infiltration, resistance to therapy, and a poor prognosis. Despite standard treatments, survival remains only approximately 14 months. Cannabinoids have been increasingly investigated for their therapeutic potential in gliomas, particularly GBM. Although multiple reviews on this field of research have been published, most are current only up to 2022. This systematic review aims to provide an updated summary of studies published between 2022 and 2025, capturing recent developments in anti‐glioma mechanisms, combinational strategies, immune modulation, and novel therapeutic platforms. Following PRISMA guidelines, PubMed, Scopus, ScienceDirect, and SpringerLink were searched for original English‐language journal articles published between January 2022 and February 2025, using search terms related to cannabinoids and brain cancer. From 1031 records, 45 original research articles were included after removing duplicates, non‐primary studies, and irrelevant topics. The studies were categorized into seven thematic domains based on content. Recent studies have elaborated on the anti‐cancer mechanisms of cannabinoids beyond endocannabinoid signaling via the CB_1_/CB_2_ receptor, including ferroptosis induction, mitochondrial dysfunction, integrated stress response activation, and epigenetic modulation. Synthetic cannabinoids and their analogs demonstrated enhanced blood–brain barrier penetration and cytotoxicity in glioma models. Cannabinoids have been shown to modulate immune responses in glioma, influencing T cell infiltration, myeloid suppressor cell recruitment, and tumor‐associated macrophage function. Novel formulation and delivery strategies have improved cannabinoid solubility, stability, and tumor targeting. Combination therapies, particularly cannabidiol with temozolomide or radiotherapy, exhibited additive or synergistic anti‐tumor effects, although variability between glioma subtypes suggests the need for personalized approaches. Although cannabinoid‐based glioma research has expanded our understanding of the mechanisms, discrepancies between preclinical findings and clinical data highlight the need for rigorous clinical trials and mechanistic research before cannabinoid‐based treatments can be reliably integrated into standard glioma care.

## Graphical abstract

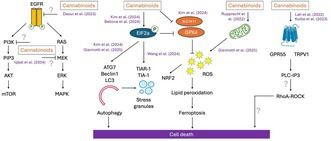

## 1 Introduction

Glioma, the cancer of glial cells, accounts for approximately 80% of all malignant primary brain tumors [1]. This disease is classified into three major subtypes: astrocytoma, oligodendroglioma, and ependymoma, based on the cellular origin [2]. The World Health Organization (WHO) classifies gliomas into grades I–IV based on their histological features and malignancy. The grade IV astrocytoma, glioblastoma (GBM), is the most aggressive and prevalent form of malignant brain tumor in adults, with a median survival of only about 14 months from diagnosis [3].

Currently, the standard treatment for GBM involves surgical resection, followed by concurrent radiotherapy and temozolomide (TMZ) chemotherapy [4]. Temozolomide is an oral alkylating agent that exerts its cytotoxic effects by methylating DNA at the O6, N7, and N3 positions of guanine residues, ultimately inducing DNA damage and apoptosis [5]. However, the therapeutic efficacy of TMZ is largely determined by the activity of O6‐methylguanine‐DNA methyltransferase (MGMT), an enzyme that repairs alkylated guanine, thereby counteracting the cytotoxic effects of TMZ and conferring chemoresistance [6]; although this correlation may be more complicated than previously believed [7].

**FIGURE 1:**
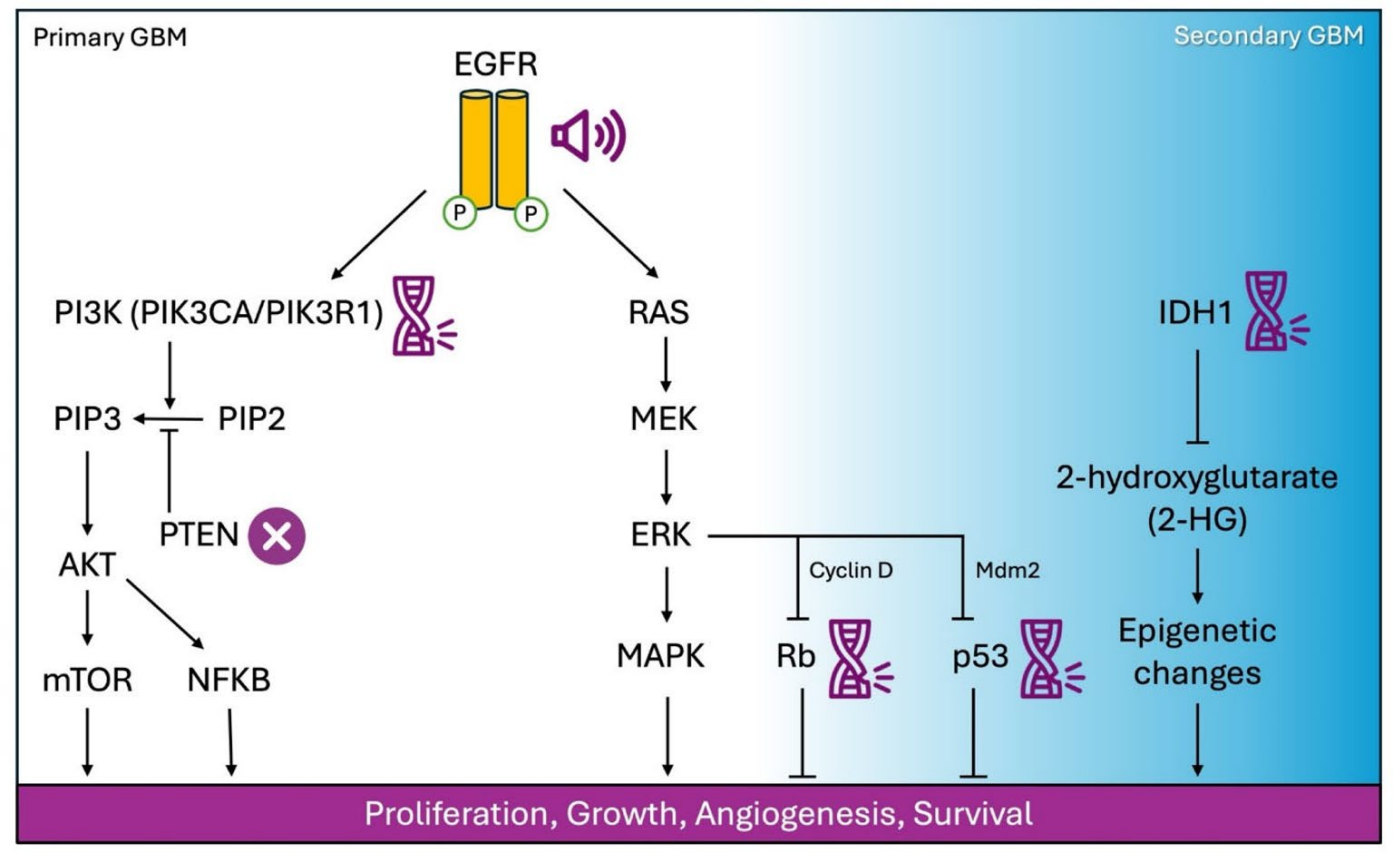
Common molecular alterations in primary and secondary glioblastoma (GBM). This schematic highlights the key oncogenic signaling pathways and mutations commonly associated with primary (left, white background) and secondary GBM (right, blue background).

GBM exhibits substantial genetic and molecular heterogeneity, with distinct subpopulations of tumor cells exhibiting variable sensitivity to therapy [8]. Primary GBM (~90% of cases) is commonly characterized by the amplification of the epidermal growth factor receptor (EGFR) [9] and deletion of the phosphatase and tensin homolog (PTEN) [10], while secondary GBM (progressed from lower grade gliomas) is more often associated with mutations of the Tumor Protein p53 (TP53) and isocitrate dehydrogenase 1 (IDH1) [11, 12] (Figure 1). Among these key genetic alterations, EGFR amplification plays a key role in GBM oncogenesis, with increasing age‐associated nuclear factor kappa‐light‐chain‐enhancer of activated B cells (NF‐κB)‐driven inflammation further exacerbating EGFR dysregulation [13]. EGFR signaling activates downstream oncogenic cascades, particularly the PI3K–AKT–mTOR and RAS–RAF–ERK pathways [14, 15]. PTEN loss leads to unchecked PI3K–AKT–mTOR activity, further enhancing the oncogenic effects of EGFR activation [16].

Other genetic alterations within the PI3K–AKT–mTOR pathway, such as mutations in phosphatidylinositol‐4,5‐bisphosphate 3‐kinase catalytic subunit alpha (PIK3CA) and phosphoinositide‐3‐kinase regulatory subunit 1 (PIK3R1), are also prevalent in GBM and contribute to its aggressive phenotype [17]. Furthermore, the loss of cell cycle control through inactivation of tumor suppressor genes, particularly the RB transcriptional corepressor 1 (RB1), promotes uncontrolled proliferation and tumor progression [18, 19]. Another challenge is posed by glioblastoma stem‐like cells (GSCs), a subpopulation of tumor‐initiating cells that exhibit intrinsic resistance to both chemotherapy and radiotherapy [20]. Beyond genetic and cellular resistance, the blood–brain barrier (BBB) significantly limits the penetration of therapeutic agents into the brain, restricting drug bioavailability and further complicating the treatment of GBM [21].

Given these limitations, there is an urgent need for novel treatment strategies for glioblastoma (GBM). Drug repurposing, the strategy of identifying new therapeutic applications for existing drugs, has gained significant traction in oncology as a cost‐effective and time‐efficient alternative to traditional drug development [22]. Natural products have long been a cornerstone of anticancer drug discovery, exhibiting diverse mechanisms of action, including modulation of cell cycle progression, induction of apoptosis, inhibition of angiogenesis, and immune system activation [23]. Given the complex molecular landscape of GBM and its resistance to conventional chemotherapies, there is growing interest in exploring naturally derived compounds as potential therapeutic agents.

**FIGURE 2:**
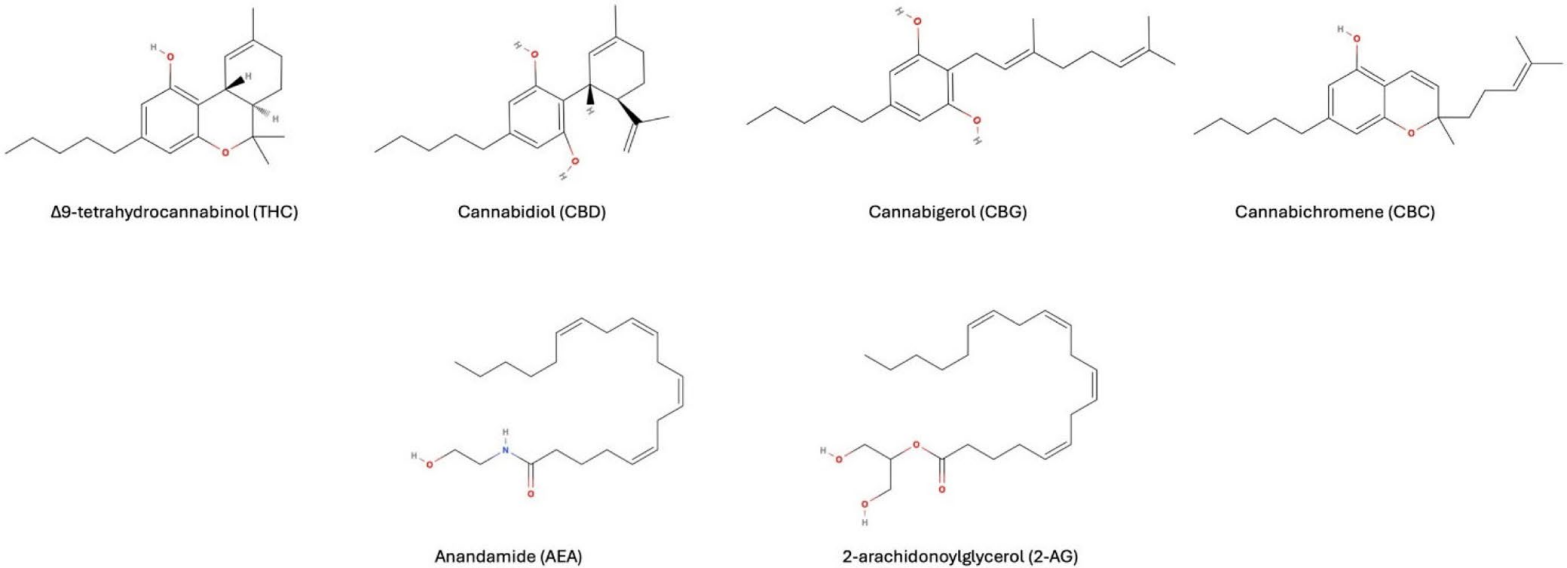
Chemical structures of some common phyto‐ and endocannabinoids.

Among these, compounds derived from the *Cannabis sativa L*. plant have attracted particular attention. This plant contains more than 150 known phytocannabinoids, with Δ9‐tetrahydrocannabinol (THC) and cannabidiol (CBD) being the most extensively studied [24]. In addition to these major constituents, lesser known cannabinoids, such as cannabigerol (CBG), cannabichromene (CBC), and their acidic precursors (e.g., CBDA, CBGA) have demonstrated promising anti‐tumor activities. Beyond plant‐derived compounds, endocannabinoids such as anandamide (AEA) and 2‐arachidonoylglycerol (2‐AG), as well as synthetic analogs, have expanded the pharmacological scope of cannabinoid‐based therapeutics [25]. These compounds signal through the cannabinoid receptor type 1 (CB1R) and the cannabinoid receptor type 2 (CB2R) of the endocannabinoid system (ECS) [26]. Despite their structural diversity (Figure 2), these ligands share key physicochemical characteristics, such as high lipophilicity and the presence of flexible aliphatic chain cores, that allow them to interact with the lipid‐rich binding pockets of CB1R and CB2R. The broad ligand specificity of these G‐protein‐coupled receptors enables them to accommodate structurally distinct molecules through conformational plasticity and multiple binding modes [27]. This promiscuity of receptor–ligand interactions allows endogenous and exogenous cannabinoids to modulate ECS signaling across a wide range of biological contexts. CB1R is highly expressed in the central nervous system, particularly in the basal ganglia, cerebellum, hippocampus, and cortex, and mediates the psychoactive effects of THC [28]. On the contrary, CB2R is primarily expressed in immune cells, suggesting a role in immunomodulation [29]. Notably, upregulation of CB2R has been observed in gliomas, particularly in higher‐grade tumors, implicating the ECS in glioma progression and immune evasion [30]. THC acts as a partial agonist at both CB1R and CB2R, while CBD exhibits a more complex pharmacological profile, acting as a negative allosteric modulator of CB1R and indirectly activating CB2R through alternative targets, including G protein‐coupled receptor 55 (GPR55), transient receptor potential vanilloid 1 and 2 (TRPV1/2), and 5‐hydroxytryptamine receptor 1A (5‐HT1A) [31, 32].

Preclinical research has shown that cannabinoids exhibit anti‐proliferative, pro‐apoptotic, and anti‐inflammatory properties in GBM through multiple mechanisms, including mitophagy induction, disruption of mitochondrial function, calcium influx through TRPV channels, and accumulation of oxidative stress [33]. Furthermore, cannabinoids influence GSCs by modulating transcription factors such as the inhibitor of DNA binding protein 1 (ID1) and acute myeloid leukemia 1 (AML‐1) [34, 35]. The ability of cannabinoids to sensitize glioma cells to chemotherapy has also been demonstrated, with CBD enhancing TMZ cytotoxicity through TRPV2 activation, while THC‐CBD combinations synergistically improve treatment responses in xenograft models [34, 36, 37]. Furthermore, a preliminary clinical trial involving the intratumoral THC administration in patients with recurrent GBM suggested reduced tumor cell proliferation, supporting the potential translational relevance of cannabinoid‐based therapies [38]. A Phase Ib randomized trial that evaluated nabiximols, an oromucosal THC‐CBD spray, in combination with TMZ for recurrent GBM also reported tolerability and potential survival benefits, but the small sample size limited its statistical power [39].

Amid these encouraging findings, there has been a rapid increase of research on cannabinoids as therapeutic candidates for cancer. The number of articles published on the topic of “cannabinoids AND cancer” has risen markedly, from 678 publications between 2015 and 2020 to 1236 between 2021 and 2024 (PubMed). To keep pace with this rapidly expanding literature, several systematic reviews have recently examined the role of cannabinoids in gliomas [40, 41, 42, 43, 44, 45], but they predominantly cover studies published before 2022. Since then, substantial progress has been made in this field. This review aims to provide an updated summary of the latest advances (2022–2025) in the field, with a focus on mechanistic insights, combination strategies, immune modulation, and innovations in cannabinoid delivery.

## 2 Methods

This systematic review was conducted in accordance with the Preferred Reporting Items for Systematic Reviews and Meta‐Analyses (PRISMA) guidelines [46]. A comprehensive literature search was performed using PubMed, Scopus, ScienceDirect, and SpringerLink to identify studies investigating the therapeutic potential of cannabinoids in gliomas published between January 2022 and February 2025. The search strategy incorporated Medical Subject Headings (MeSH) terms and free text keywords to ensure broad coverage of the relevant literature. The final search query was: “cannabinoid” [MeSH Terms] OR “cannabinoids” [MeSH Terms] OR cannabinoid OR cannabinoids OR cannabidiol OR THC OR CBD AND (brain cancer OR glioma OR glioblastoma) AND (“2022/01/01”[Date—Publication]: “2025/12/31”[Date—Publication]) AND (journal article[Publication Type] AND English[Language]).

**FIGURE 3:**
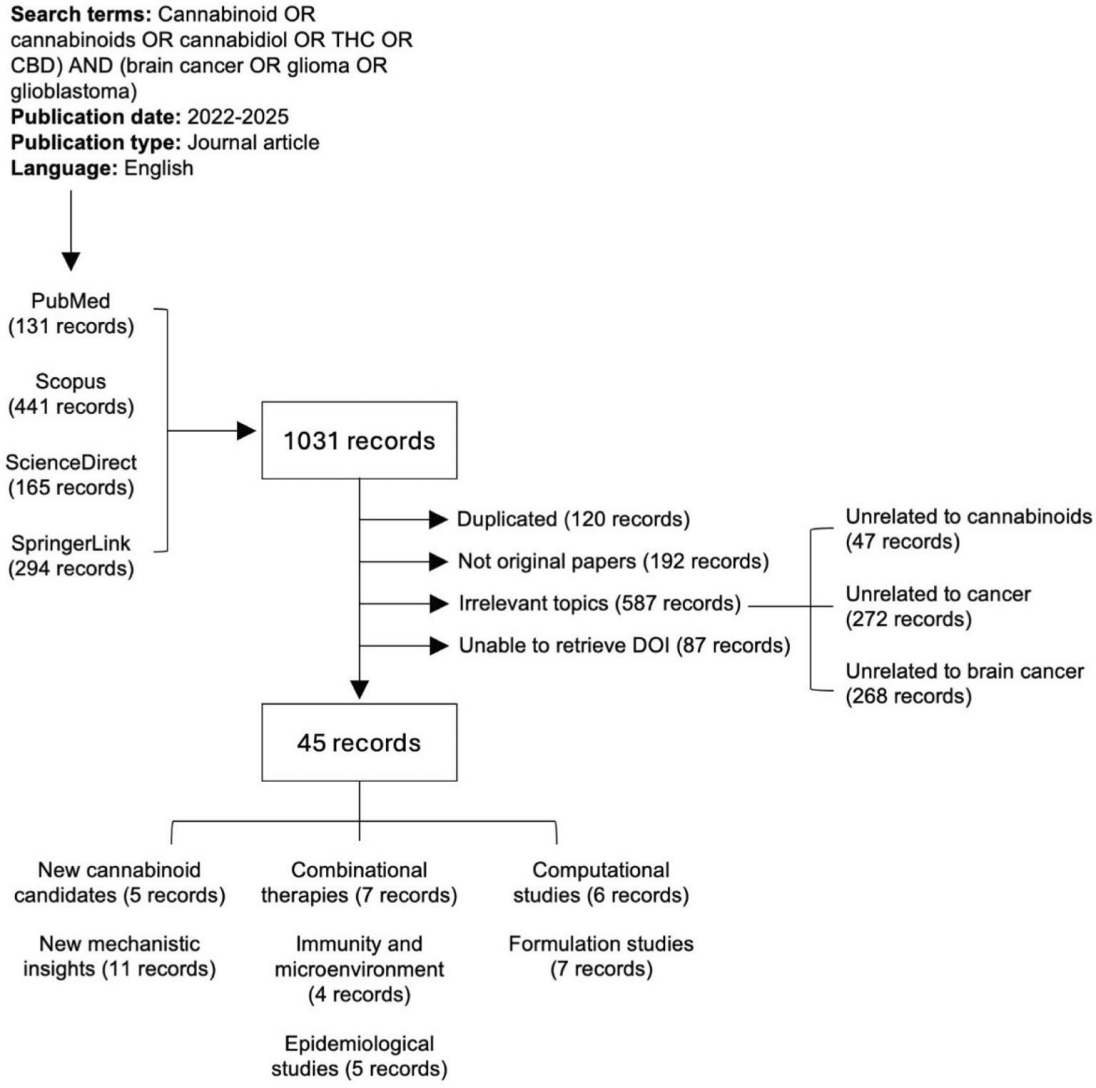
PRISMA‐based flow diagram of study selection for cannabinoid and brain cancer research (2022–2025).

The search was restricted to peer‐reviewed journal articles in English. No restrictions were placed on the study design to ensure a comprehensive review of both preclinical and clinical research. The retrieved records were processed using R version 4.3.0 (2023‐04‐21) and relevant R packages, including rentrez, revtools and PRISMA2020, supplemented with additional manual cataloguing. A total of 1031 papers have been retrieved, of which 120 duplicated records have been removed. Studies were included if they investigated the role of cannabinoids (e.g., THC, CBD) in gliomas, including glioblastoma (GBM), diffuse midline glioma (DMG), astrocytomas, oligodendrogliomas, or ependymomas, reported original research (preclinical or clinical) on cannabinoids as therapeutic agents in gliomas, and evaluated the effects of cannabinoids either alone or in combination with other therapies (e.g., chemotherapy, radiotherapy, immunotherapy). Studies were excluded for the following reasons: Not original articles (e.g., reviews or commentaries, *n* = 192), not related to cannabinoids (e.g., studies on cannabinoid receptor biology without cannabinoid treatment, *n* = 47), not related to cancer research (e.g., studies focused on the neurological effects of cannabinoids, *n* = 272), not related to gliomas or brain cancers (*n* = 268) and unable to retrieve DOI (*n* = 87). The remaining 45 articles were included in this systematic review. A PRISMA flow diagram (Figure 3) was generated to summarize the selection process and details of all screened records are available in Table S1.

## 3 Results

This systematic review identifies five key areas of advancement in cannabinoid‐based glioma research since 2022: (1) bioinformatic drug prediction, (2) mechanistic insights into cannabinoid action, (3) tumor microenvironment and immune modulation, (4) cannabinoid delivery systems, (5) combination therapy strategies.

### 3.1 Bioinformatic Predictions Suggest Cannabinoids as Potential Anti‐GBM Candidates

Recent bioinformatic studies have begun to identify cannabinoid‐related pathways as potential therapeutic targets in gliomas, offering an unbiased foundation for the therapeutic potential of cannabinoids. For example, through large‐scale single‐cell RNA sequencing analyses in GBM tumors, Lee et al. [47] identified CB1R as one of the “primary target genes,” defined as GBM‐specific genes that are known pharmacological targets and exhibit consistent expression across patient samples (i.e., low inter‐patient variability). Interestingly, this group screened 132 repurposed drugs, including 67 neuroactive agents (NADs) and 65 oncology drugs, using ex vivo tumor culture and identified Rimonabant, a CB1R antagonist, as a top‐ranked NAD (rank 6) for cytotoxicity of GBM. Also using a transcriptomic approach, Yang et al. [48] screened 1161 genes associated with programmed cell death (PCD) in 512 low‐grade glioma (LGG) samples and found that high expression of genes involved in the ECS pathway, including CB1R and CB2R, was associated with a better prognosis. Furthermore, Kishk et al. [49] constructed patient‐specific metabolic models from transcriptomic data from The Cancer Genome Atlas (TCGA) to simulate GBM tumor metabolism. They identified a potential synergy between CBD and adapalene, a selective agonist of retinoic acid receptors (RARs), in targeting GBM. Using the Glioblastoma‐based Biomedical Profile Network (GBPN), a large‐scale integrative network derived from the NCATS GARD Knowledge Graph of diseases, genes, and drugs, McGowan et al. [50] revealed putative regulatory nodes relevant to GBM. Cannabinoids are known to modulate several of the pathways highlighted in this analysis, including cell cycle regulation, oxidative stress response, and immune signaling, suggesting that CBD may be an effective drug candidate against GBM and related diseases.

Together, these studies consistently highlighted ECS‐related pathways as prognostically significant and pharmacologically actionable targets across different glioma subtypes, laying a systems‐level foundation for the development of cannabinoids as glioma therapy.

### 3.2 New Mechanistic Insights Into the Anti‐Glioma Effects of Cannabinoids

**FIGURE 4:**
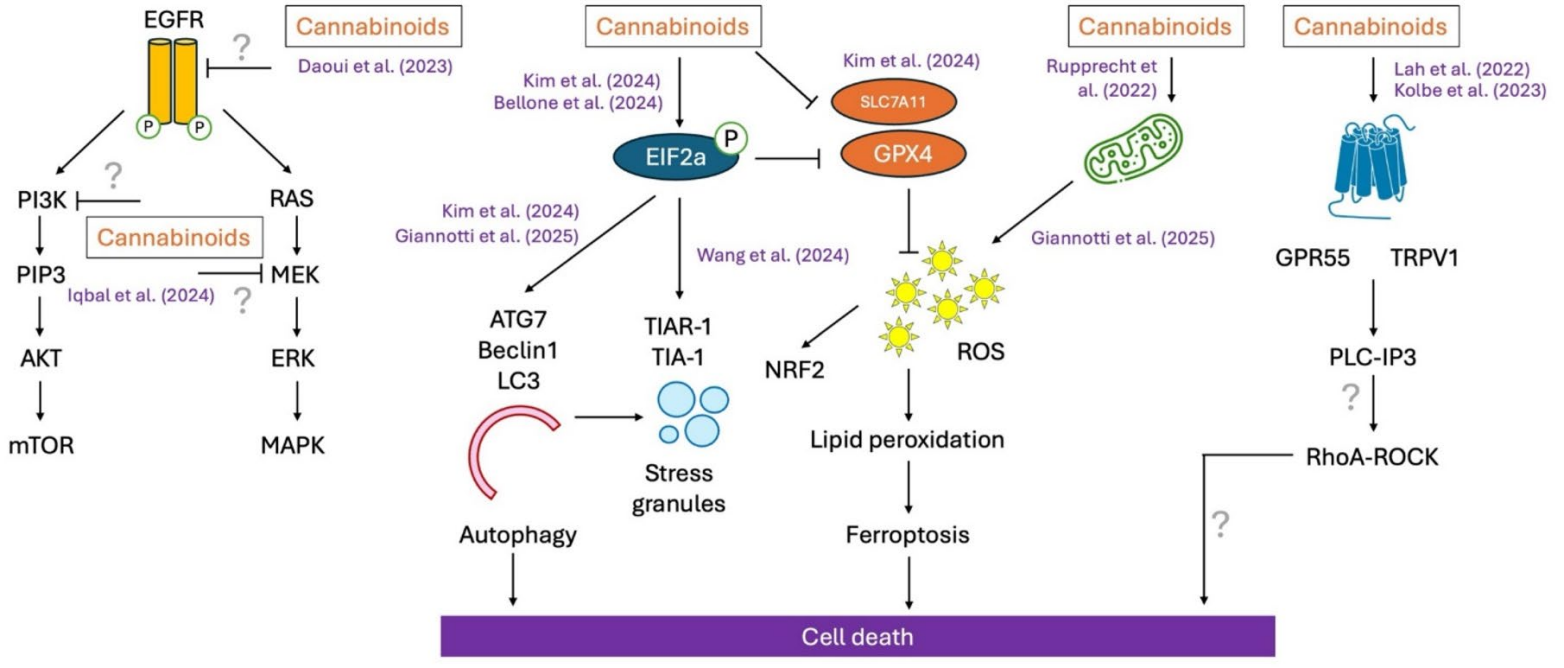
Emerging mechanisms of cannabinoid‐induced cell death in glioma.

Recent studies have further elucidated the molecular mechanisms by which cannabinoids exert anti‐glioma effects, revealing new targets and pathways involved in cell death and tumor suppression (Figure 4). Kim et al. [51] and Giannotti et al. [52] confirmed previous observations that CBD treatment increases the expression of autophagy markers, such as Beclin‐1 and LC3‐II, and activates the NRF2 pathway, a master regulator of the response to oxidative stress. In addition, CBD treatment led to suppression of GPX4 and SLC7A11 in the U87 and U373 GBM cell lines, molecular hallmarks of ferroptosis. Ferroptosis is a form of regulated cell death characterized by iron‐dependent lipid peroxidation, leading to oxidative membrane damage and cell death [53]. The induction of ferroptosis aligns with the broader cytotoxic profile of cannabinoids, which have been shown to disrupt redox homeostasis and induce oxidative stress.

Bellone et al. [54] used a combination of proteomics and pulse‐SILAC metabolic labeling to demonstrate that CBDA induces eIF2α phosphorylation, leading to an accumulation of ubiquitinated proteins and autophagosomes. eIF2α, a translation initiation factor, plays a key role in the synthesis of stress‐responsive proteins, and its activation is a hallmark of the integrated stress response (ISR) and translational reprogramming in tumor cells [55]. Wang et al. [56] further demonstrated that CBD significantly increases the size of TIAR‐1 positive stress granules (SGs) in tumor cells, known to be a downstream phenotype of eIF2α activation. These studies corroborate earlier findings by Salazar et al. [57], which showed that cannabinoids induce endoplasmic reticulum (ER) stress in GBM.

While the anti‐tumor potential of cannabinoids in GBM has traditionally been attributed to the modulation of ECS receptors, the roles of GPR55 and TRPV1 have also been the focus of recent investigation. Lah et al. [58] showed that CBD and CBG, in a 3:1 M ratio (CBD:CBG), can inactivate GPR55 signaling and induce significant cytotoxicity in patient‐derived GBM cells, with particularly strong effects in GSCs. Additionally, TRPV1, a calcium‐permeable ion channel, was shown to become desensitized with cannabinoid treatment, leading to calcium dysregulation, endoplasmic reticulum (ER) stress, and cancer cell death. In contrast, Kolbe et al. [59] reported that THC activated GPR55, leading to reduced proliferation, as indicated by decreased Ki67 immunoreactivity, in patient‐derived GBM cells. This effect was mediated through PLC‐IP3 and RhoA‐ROCK signaling. These apparent inconsistent findings may reflect specific cell‐type signaling or ligand‐dependent biased agonism, whereby different cannabinoids stabilize distinct GPR55 conformations and preferentially engage different intracellular pathways. Such findings underscore the pharmacological complexity of cannabinoid receptors in glioma.

Emerging studies are beginning to reveal alternative molecular targets and signaling pathways through which cannabinoids can exert their effects. Rupprecht et al. [60] demonstrated that treatment with THC and CBD leads to a marked reduction in the oxygen consumption rate and ATP production, possibly due to down‐regulation of key respiratory chain proteins, such as subunits of complexes I and IV of the electron transport chain. In particular, this effect is not reversed by established cannabinoid‐induced cell death pathways, such as CB1R‐ and CB2R‐mediated autophagy, suggesting that mitochondrial impairment induced by THC and CBD occurs through a previously unknown mechanism. Some non‐canonical cannabinoid targets have been proposed by computational modeling approaches. Daoui et al. [61] conducted in silico docking studies of 50 cannabis‐derived phytochemicals (12 cannabinoids and 38 terpenes) against EGFR, a key oncogenic driver of GBM. They reported that seven cannabinoids (CBDA, CBD, THCV, CNB, CBL, Δ‐9‐THC, and Δ‐8‐THC) exhibited comparable or even superior binding affinity relative to standard EGFR inhibitors Erlotinib and Tamoxifen. Using a similar computational approach, Iqbal and Matsabisa [62] predicted the binding of cannabinol and THC to additional key signaling proteins, including MEK and PI3K. Further studies are needed to experimentally validate these interactions and clarify their role in GBM cell death induced by cannabinoids.

Diffuse midline glioma (DMG), a highly aggressive pediatric brain tumor, is characterized by the K27M mutation of the histone H3 gene, which drives widespread epigenetic dysregulation. A key downstream effector is ID1, a transcriptional regulator associated with tumor cell invasion and stem‐like behavior. Messinger et al. [63] demonstrated that CBD treatment suppresses ID1 expression in DMG cells by reducing H3K27ac enrichment at the ID1 locus. Functional assays, including wound healing and Transwell migration experiments, confirmed that ID1 downregulation correlates with decreased tumor cell motility. These findings suggest that CBD may exert its anti‐tumor effects not only through receptor‐mediated signaling but also by reprogramming epigenetic states in glioma cells.

### 3.3 The Impact of Cannabinoids on the Tumor Microenvironment

Recent studies have increasingly recognized the tumor microenvironment (TME) as a critical determinant of the progression of glioma, immune evasion, and therapeutic resistance [64]. Cannabinoids have emerged not only as cytotoxic agents but also as modulators of the TME. In a landmark study, Zhou et al. [65] showed that CBD, delivered by nanoparticles called “Nano‐reshaper” (see below) enhanced systemic T‐cell proliferation and countered GBM‐induced lymphopenia in a GL261 orthotopic murine GBM model. Khodadadi et al. [66] also demonstrated that inhaled CBD significantly suppressed tumor growth in a similar mouse model by increasing CD8^+^ T cell infiltration while reducing the abundance of innate lymphoid cells (ILCs), collectively promoting a more immunostimulatory tumor environment. In addition, this group showed that CBD reduced the expression of pro‐angiogenic and pro‐inflammatory mediators such as P‐selectin, apelin, and IL‐8, while downregulating the immunosuppressive enzyme IDO1.

The role of CB2R as a possible immunomodulating mechanism of CBD has been investigated. Duan et al. [67] reported that pharmacological inhibition of CB2R using AM630 resulted in tumor regression, increased CD8^+^ T cell infiltration, and upregulation of cytotoxic markers granzyme B and perforin. On the contrary, activation of CB2R with GW405833 promoted tumor progression and immunosuppression. However, the findings of Lu et al. [68] offered a contrasting view: activation of CB2R by a different agonist, JWH133, improved tumor‐associated macrophage‐mediated phagocytosis of glioma cells. These divergent findings suggest that CB2R ligands may exhibit biased signaling, with different agonists triggering different immune outcomes depending on the cellular context and downstream pathway activation.

Beyond immune cells, cannabinoids can also interfere with interactions between GBM and stromal components of the TME. Hohmann et al. [69] used 3D co‐culture spheroid models to show that CBD and THC increased tumor spheroid size in glioma and melanoma cells; although no significant changes in proliferation or apoptotic markers were observed. This phenomenon may reflect cannabinoid‐induced alterations in GBM cell adhesion or motility and could be abolished when spheroids were co‐cultured with astrocytes or microglia. These glial cells were shown to inhibit collective migration of GBM cells through the release of secretory factors. Furthermore, Williams et al. [70] suggested that different regions of the GBM tumor could respond to cannabinoids differently, possibly due to different receptor expression profiles and transcriptional landscapes. Their study showed that U87 cells, a widely used glioblastoma cell line originally derived from a human GBM tumor, exhibited widespread transcriptional changes to the CB2R inverse agonist AM630, including suppression of cell cycle regulators and activation of immune‐related pathways such as TP53 and interferon signaling. On the contrary, GIN‐8 cells, a patient‐derived cell line isolated from the invasive tumor margin, displayed fewer transcriptional changes, likely due to low expression of CB2R and CB1R. These findings underscore the spatial heterogeneity of GBM and imply that cannabinoid‐based therapies may need to be tailored not only to the tumor subtype but also to the specific anatomical and molecular characteristics of different tumor zones.

Taken together, these findings suggest that cannabinoids, particularly CBD, exert multifaceted effects on the microenvironment of GBM. However, the variability in response influenced by the anatomical context and migration characteristics of GBM cells calls for further mechanistic studies to define the therapeutic strategy for cannabinoids in GBM.

### 3.4 Advances in Formulations That Improve the Delivery of Cannabinoids

While the therapeutic potential of cannabinoids in the treatment of GBM has been increasingly recognized, their clinical translation remains hindered by poor bioavailability, rapid metabolism, and limited penetration across the BBB. To address these limitations, researchers have developed various nanoparticle‐mediated delivery systems to enhance the pharmacokinetic properties of cannabinoids. A widely explored strategy involves the use of polymer‐coated nanoparticles (PCNPs). For example, Kuźmińska et al. [71] developed PLGA‐based nanoparticles co‐loaded with cannabidiol (CBD) and etoricoxib, a selective COX‐2 inhibitor. The resulting nanoparticles exhibited a small size (< 400 nm), a high encapsulation efficiency (78.43% for CBD) and a sustained drug release profile, along with enhanced cytotoxicity in GBM cell lines (T98G and U‐138). Nanoemulsions (NEs) represent another class of delivery systems designed to enhance the oral and systemic bioavailability of cannabinoids. NEs are colloidal oil‐in‐water dispersions, typically 20 to 200 nm in size, stabilized by surfactants. They improve solubility, stability, and BBB penetration, and can offer sustained drug release. Mobaleghol Eslam et al. [72] compared bulk THC/CBD, empty NEs, and drug‐loaded NEs (NEDs) in a rat GBM model, showing a four‐fold reduction in tumor volume in rats treated with NED. Borges et al. [73] developed a multi‐charged NE system incorporating CBD, indocyanine green (ICG), and protoporphyrin IX (PpIX) for photodynamic therapy (PDT). This NE improved intracellular uptake and tumor selectivity, with near‐infrared irradiation (810 nm) inducing significant tumor reduction in 2D/3D in vitro models and an orthotopic mouse model of GBM, supporting the feasibility of combinatorial cannabinoid‐PDT strategies.

Muresan et al. [74] compared the biodistribution of CBD delivered by intrathecal injection of PCNPs and NEs and observed that PCNPs enabled faster transport of CBD to the brain (*T*_max_ = 30 min) but are cleared more quickly, leading to lower accumulation, while NEs resulted in slower *T*_max_ (120 min) but higher CBD retention in the brain over 4 h, suggesting that these two formulations can be tailored to desired pharmacokinetic outcomes (e.g., rapid vs. sustained effect).

Beyond NEs and PCNPs, several other nanoparticle‐based and solid‐state systems have emerged. Hatziagapiou et al. [75] used cyclodextrin (CD) inclusion complexes to improve CBD solubility and in vitro anticancer efficacy. CDs are cyclic oligosaccharides with a hydrophobic interior cavity and hydrophilic exterior, allowing CBD encapsulation while maintaining aqueous dispersibility. Stasiłowicz‐Krzemień et al. [76] used a codispersion system comprising Soluplus (an amphiphilic polymer) and Neusilin US2 (a porous adsorbent) to deliver a crude supercritical CO_2_ extract of *Cannabis sativa* (Henola variety), containing CBD, CBDA, and CBC. The parallel artificial membrane permeability assay (PAMPA) showed a high BBB penetration potential, and the solid‐state formulation exhibited Fickian diffusion kinetics, offering improved stability and oral delivery potential compared to liquid NEs. Zhou et al. [65] developed an inorganic–lipid hybrid nanoparticle system called Nano‐shaper, comprising a calcium phosphate core (CaP), which enables pH‐responsive drug release, and a lipid coating for the co‐delivery of CBD and the immunostimulatory cytokine LIGHT. These nanoparticles were functionalised with Apolipoprotein E (ApoE) to facilitate BBB penetration.

In addition to systemic delivery strategies, researchers have explored localized delivery platforms to bypass the BBB and directly target the tumor site. Muresan et al. [77] developed a biodegradable microneedle‐based patch designed for implantation at the post‐surgical tumor resection site. The patch was loaded with PEG–PLA‐coated nanocrystals of CBD and the poly(ADP‐ribose) polymerase (PARP) inhibitor olaparib (OLA), allowing sustained drug release into the surrounding brain parenchyma. The nanocrystal formulation significantly improved CBD solubility and stability, achieving 59.6 μg/g tissue concentration within 30 min of insertion in the ex vivo brain. The diffusion of OLA reached depths of up to 6 mm, and the microneedles exhibited sufficient mechanical strength (> 0.68 N/needle) for effective penetration into brain tissue. This solid‐state, localized approach offers a promising strategy for eliminating residual GBM cells following surgical resection, especially for hydrophobic agents such as CBD with poor systemic delivery profiles.

### 3.5 Cannabinoid‐Based Combination Therapy for GBM

Both in vitro and in vivo studies have suggested that cannabinoids, especially CBD and THC, can sensitize GBM cells to TMZ. A Phase II trial, known as the ARISTOCRAT study, is currently underway to expand an earlier clinical study [39] to evaluate the efficacy of nabiximols (a 1:1 mixture of THC and CBD) in combination with TMZ in patients with MGMT‐methylated recurrent GBM [78]. If successful, this study could provide the first robust clinical evidence supporting the preclinical findings.

New findings have provided a possible molecular explanation for the benefit of cannabinoid‐induced chemosensitization. Using patient‐derived GBM models and orthotopic xenografts, Soroceanu et al. [79] demonstrated that CBD inhibits RAD51, a key mediator of homologous recombination (HR)‐mediated DNA repair, making GBM cells more susceptible to DNA damage. Furthermore, Brookes et al. [80] identified a novel epigenetic mechanism underpinning CBD's synergy with TMZ. Using 3D OrbiSIMS mass spectrometry, the study revealed that CBD and its fluorinated analog (4′‐F‐CBD) induce DNA methylation on the CpG islands, potentially affecting the expression of DNA repair enzymes such as MGMT through promoter hypermethylation. Interestingly, CBD is known to reduce global DNA methylation in the mouse hippocampus [81, 82], suggesting the epigenetic effects of CBD appear to be highly context dependent.

Although the combination of cannabinoids and TMZ has gained significant attention, Załuska‐Ogryzek et al. [83] explored the effects of combining CBD and AM1172, another ligand of CB2R, with cisplatin. Interestingly, while AM1172 synergised with cisplatin in neuroblastoma cells, a cancer of the sympathetic nervous system, it showed an antagonistic effect in C6 rat glioma cells. Similarly, CBD demonstrated an additive interaction with cisplatin in most GBM models, but in T98G cells, the combination was antagonistic. These findings underscore tumor heterogeneity as a critical determinant of cannabinoid–chemotherapy efficacy and suggest that patient‐specific molecular profiling may be necessary to optimize treatment regimens.

The synergy between cannabinoids and radiotherapy represents another emerging therapeutic avenue. Chan et al. [84] examined the radiosensitizing potential of beta‐caryophyllene (BCP), a natural cannabinoid, in U87MG and GL261 cell lines, as well as an orthotopic murine GBM model. The study showed that BCP enhances radiotherapy efficacy by delaying DNA damage repair and sustaining γH2AX foci formation. PPARγ and NF‐κB signaling pathways were significantly modulated, resulting in increased apoptosis and impaired survival signaling by a reduction in the activity of pERK and pAKT. These findings suggest that cannabinoid‐based radiosensitizers could improve radiotherapy outcomes, particularly by targeting DNA repair mechanisms.

### 3.6 Novel Synthetic Cannabinoids as Potential Therapeutic Candidates

Recent advances in synthetic chemistry have enabled the design of structurally novel cannabinoid analogs that bypass the need for plant extraction. This not only avoids variability in phytochemical composition and the presence of psychoactive contaminants such as THC, but also circumvents regulatory and legal constraints associated with handling controlled cannabis‐derived substances. Moreover, synthetic approaches allow for precise control over ligand structure, enabling optimization of receptor selectivity, metabolic stability, and pharmacokinetic profiles [85].

**FIGURE 5:**
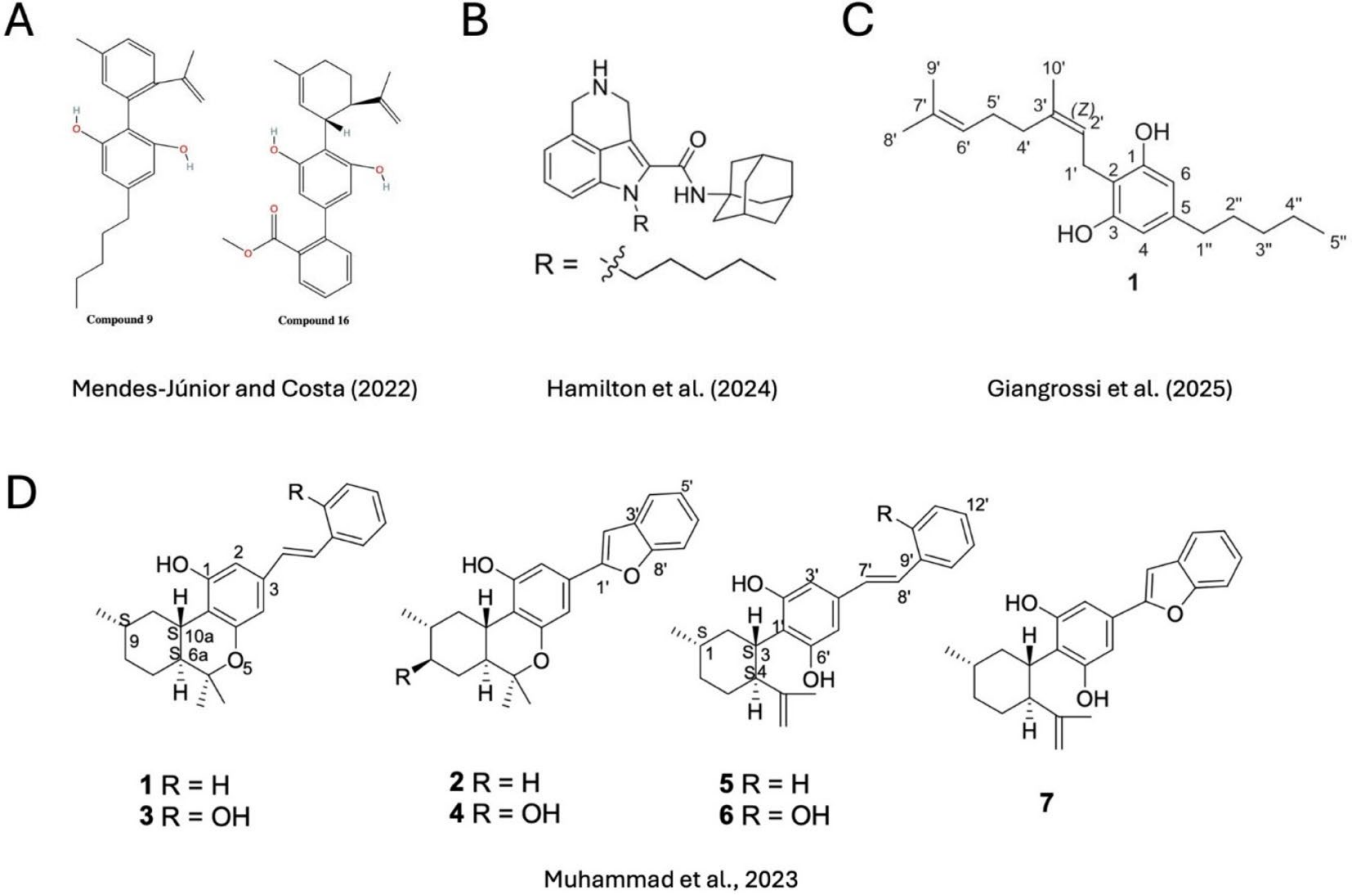
Structures of recently synthesized natural and synthetic cannabinoids with potential anticancer activities.

Several recent studies have adopted rational drug design strategies to create next‐generation cannabinoid‐inspired molecules with improved therapeutic properties. Mendes Júnior and Costa [86] generated 16 novel CBD analog through systematic modifications of side chains and functional groups. These compounds were computationally evaluated for oral bioavailability, blood‐brain barrier permeability, receptor affinity, cytochrome P450 interaction, and predicted toxicity using tools such as SwissADME and OSIRIS. Two candidates, known as compounds 9 and 16 (Figure 5A), exhibited favorable pharmacological properties, including high affinity for CB1R and CB2R and improved BBB penetration, without predicted mutagenicity or hepatotoxicity. In particular, compound 9 was predicted to cross theblood‐brain barrier, making it a promising candidate for central nervous system diseases such as glioblastoma.

Hamilton et al. [87] synthesized a new series of tricyclic indole‐2‐carboxamides using rational drug design, aiming to improve the therapeutic profile of cannabinoid‐like compounds for pediatric gliomas. These molecules were structurally inspired by WIN 55212‐2, a well‐known indole‐based synthetic cannabinoid, and featured modifications at the C‐4 position of the indole scaffold to introduce a rigid six‐membered nitrogen‐containing ring. These structural modifications were intended to improve lipophilicity, conformational restriction, and metabolic stability. Six final compounds were synthesized and evaluated for cannabinoid receptor activity and cytotoxicity in KNS42 pediatric GBM cells. Interestingly, transcriptomic profiling of KNS42 cells treated with the lead compound, named Compound 23 (Figure 5B) revealed downregulation of the FOXM1 cell cycle network, suggesting a CB‐independent mechanism involving disruption of mitotic gene expression and ROS‐related stress pathways.

Giangrossi et al. [88] employed a biomimetic approach to synthesize rare CBG‐type cannabinoids, which are typically present only in trace amounts in *Cannabis sativa* . The study involved the chemical conversion of olivetolic acid to structurally novel analog of cannabigerolic acid (CBGA), a key biosynthetic precursor for several minor cannabinoids (Figure 5C). These newly synthesized compounds were subsequently evaluated for their cytotoxic activity in GBM cell lines.

Endocannabinoid metabolites derived from sources outside of *Cannabis sativa* have also been explored as potential therapeutic candidates. One such study investigated machaeriols and machaeridiols, naturally occurring diterpenoids isolated from *Machaerium* species, which share structural similarities with classical cannabinoids [89]. The authors employed rational drug design to modify known cannabinoid scaffolds, focusing particularly on side chain alterations and functional group substitutions. A subset of the modified compounds showed moderate to high affinity for CB2R (Figure 5D). In GBM cell lines, these compounds were shown to inhibit proliferation and induce apoptosis, with effects that were further enhanced in combination with established chemotherapeutic agents. Silva et al. [90] used a biocatalytic approach to generate two fatty acid amides (FAAs), named FAA1 and FAA2, from complex oil‐derived mixtures (AO1 and AO2) extracted from *Carapa guianensis* . These FAAs exhibit binding affinity for cannabinoid receptors. Their effects were evaluated in glioma cell models, where they demonstrated dose‐dependent inhibition of cell viability and migration, while sparing normal glial cells, suggesting tumor selectivity. Mechanistically, the study identified modulation of the PI3K/AKT signaling pathway, a key driver of GBM, supporting the notion that these synthetic endocannabinoid analog may exert their anti‐cancer effects through receptor‐mediated and downstream metabolic mechanisms.

## 4 Discussion

Significant progress has been made in cannabinoid‐based glioma research in the past 3 years, advancing our understanding of its therapeutic potential and mechanistic interactions with glioma pathophysiology. Recent studies have shown the ability of cannabinoids to induce apoptosis, autophagy, and ferroptosis while revealing mechanisms such as EIF2A‐mediated proteotoxic stress, stress granule formation, and ID1 suppression. These findings expand the landscape of cannabinoid‐induced cytotoxicity beyond classical CB1R and CB2R modulation, suggesting that cannabinoids may target multiple stress response pathways in glioma cells. Preclinical studies have also discovered new roles for cannabinoids in modulating the tumor microenvironment, especially through the influences of immune cell infiltration and tumor‐associated macrophage function. Importantly, computational studies, including bioinformatic drug repurposing and transcriptomic perturbation analysis, have independently supported the potential therapeutic value of cannabinoids in glioma by identifying ECS‐related pathways as being dysregulated in glioma tissues and predictive of treatment response. Beyond mechanistic insights, translational progress has been made in optimizing cannabinoid delivery systems, with nanoparticle‐based formulations improving bioavailability, BBB penetration, and tumor selectivity. The development of novel synthetic cannabinoids has further expanded the pharmacological repertoire, introducing compounds with improved receptor specificity and metabolic stability. Meanwhile, combination therapy studies have provided evidence that cannabinoids can improve standard of care treatments, particularly in combination with TMZ and radiotherapy. However, the field still faces major challenges in bridging preclinical discoveries with clinical applications.

One of the most pressing issues remains the lack of clinical data on the efficacy and side effects of cannabinoid‐based glioma therapy. Clinical trials, such as the ARISTOCRAT study, mark an essential step towards validating preclinical findings in human patients. However, a recent clinical trial on orally administered CBD in patients with prostate, breast, colorectal, and gynecological cancers showed no significant effect on survival outcomes and tumor progression [91]. Although this study, which tracked disease progression for only up to 56 days, does not necessarily invalidate the anticancer potential of CBD, it highlights the challenge of translating preclinical findings into clinical settings. Meanwhile, cannabis use did not show a significant protective effect in terms of GBM recovery outcomes [92]. In fact, recent epidemiological analyses have reported a significant positive correlation between cannabis exposure and some cancers, including brain cancers [93, 94, 95, 96], a finding further supported by a Mendelian randomization study [97]. Although the significance of these findings, based on data on the consumption of cannabis, which contains a complex mixture of molecules and in undetermined doses, is difficult to interpret, they nevertheless raise doubts about the safety of the therapeutic use of cannabinoids. Future clinical studies must distinguish between purified cannabinoids, synthetic analog, and whole plant cannabis extracts, and should incorporate patient stratification based on molecular and immune profiles to identify subpopulations most likely to benefit from cannabinoid treatment.

Despite these challenges, the rapid evolution of cannabinoid research in gliomas highlights several promising directions for future investigation. Continued refinement of cannabinoid formulations, particularly those that incorporate targeted nanocarriers, may improve therapeutic efficacy while minimizing systemic side effects. Additionally, the integration of cannabinoids with immunotherapies represents a promising avenue for exploration, as preliminary evidence suggests that cannabinoids can influence glioma immune surveillance. Moreover, the growing interest in synthetic and semi‐synthetic cannabinoid analog provides an opportunity to fine‐tune cannabinoid pharmacology through “scaffold‐hopping”, developing compounds that maximize anti‐tumor activity while avoiding psychoactive effects or immunosuppressive properties. Importantly, mechanistic studies investigating how cannabinoids modulate cell death, metabolism, DNA repair, and immune signaling in glioma cells may uncover novel molecular targets. These targets could be explored in high‐throughput drug screening or rational drug design, potentially leading to the development of non‐cannabinoid compounds that mimic the therapeutic benefits of cannabinoids while overcoming their limitations.

In conclusion, while the past 3 years have seen significant progress in cannabinoid research for gliomas, considerable gaps remain in translating these findings into effective clinical treatments. Resolving inconsistencies between preclinical, epidemiological, and clinical data will be key to determining the true therapeutic potential of cannabinoids in gliomas. Although it is premature to consider cannabinoids as established adjuncts to glioma therapy, continued advances in drug delivery, rational cannabinoid design, and precision oncology may help clarify their role and gradually integrate them into future treatment strategies.

## Author Contributions

Farideh Javid conceptualized the study, drafted the initial version of the manuscript, and finalized the manuscript for submission. Yun Wah Lam conducted the literature review, the conceptual development and interpretation of findings, and co‐led the writing and revision of the manuscript. Andrej Belancic and Man Ki Kwok critically reviewed the manuscript and provided revisions to improve its content. All authors contributed significantly and meet ICMJE criteria for authorship. All authors read and approved the final version to be published.

## Conflicts of Interest

The authors declare no conflicts of interest.

## Supporting information


**Table S1:** Summary of all records screened between 2022 and 2025.

## Data Availability

No new data were generated. All information available is sent upon reasonable request to the corresponding author.
